# The Transplantation Resistance of Type II Diabetes Mellitus Adipose-Derived Stem Cells Is Due to G6PC and IGF1 Genes Related to the FoxO Signaling Pathway

**DOI:** 10.3390/ijms22126595

**Published:** 2021-06-19

**Authors:** Michiko Horiguchi, Yuya Turudome, Kentaro Ushijima

**Affiliations:** Division of Pharmaceutics, Faculty of Pharmaceutical Sciences, Sanyo-Onoda City University, 1-1-1 Daigaku-Dori, Sanyo Onoda 756-0884, Japan

**Keywords:** stem cell, adipose-derived mesenchymal stem cells (ADSC), type II diabetes mellitus, transplant resistance, FoxO signaling pathway, *G6PC3*, *IGF1*

## Abstract

In cases of patients with rapidly progressive diabetes mellitus (DM), autologous stem cell transplantation is considered as one of the regenerative treatments. However, whether the effects of autonomous stem cell transplantation on DM patients are equivalent to transplantation of stem cells derived from healthy persons is unclear. This study revealed that adipose-derived mesenchymal stem cells (ADSC) derived from type II DM patients had lower transplantation efficiency, proliferation potency, and stemness than those derived from healthy persons, leading to a tendency to induce apoptotic cell death. To address this issue, we conducted a cyclopedic mRNA analysis using a next-generation sequencer and identified *G6PC3* and *IGF1*, genes related to the FoxO signaling pathway, as the genes responsible for lower performance. Moreover, it was demonstrated that the lower transplantation efficiency of ADSCs derived from type II DM patients might be improved by knocking down both *G6PC3* and *IGF1* genes. This study clarified the difference in transplantation efficiency between ADSCs derived from type II DM patients and those derived from healthy persons and the genes responsible for the lower performance of the former. These results can provide a new strategy for stabilizing the quality of stem cells and improving the therapeutic effects of regenerative treatments on autonomous stem cell transplantation in patients with DM.

## 1. Introduction

Diabetes mellitus (DM) is a long-term glucose metabolic abnormality associated with hyperglycemia and/or insulin resistance [1,2,3,4]. The prevalence of DM has increased to 463 million patients, suggesting that one out of five adults aged ≥65 years suffer from DM as of 2019, according to the 9th Edition of the IDF Diabetes Atlas, which compiles the results of the latest worldwide survey of DM [5,6]. Complications, such as blindness due to diabetic retinopathy and renal failure, could be developed in patients with severe DM [7]. Accordingly, regenerative stem cell transplantation is considered for the definitive treatment of rapidly progressive and severe DM [8].

The use of various types of stem cells for regenerative therapies in patients with diabetes has been examined [9,10]. Embryonic stem cells (ESCs) were confirmed to differentiate into insulin-producing cells. However, ethical apprehension interrupts the stable supply of cells, which is a barrier to the practical use of ESCs [11,12]. Target-induced pluripotent stem (iPS) cells could be obtained stably from somatic cells [9,11]. Nevertheless, there are two problems: (1) it is time-consuming to culture cells until a sufficient amount is produced, and (2) there is apprehension about the risks of developing mutations which become cancerous in the reprogramming process [9]. Mesenchymal stem cells (MSCs) help in avoiding this problem and contribute to regenerative treatments as they secrete a large number of immune regulators and tissue-regenerating factors [13]. However, there is difficulty in controlling the differentiation of MSCs into β cells [14]. Therefore, undifferentiated MSCs are transplanted to produce cell growth factors, immune regulators, and tissue-regenerating factors.

MSCs may be obtained from various tissues; adipose-derived mesenchymal stem cells (ADSCs) and bone marrow-derived mesenchymal stem cells (BMSCs) are commonly used for regenerative treatments. ADSCs, which are contained in tissues at a higher rate than BMSCs, may be harvested in large amounts [15]. Moreover, ADSCs produce an excess of vascular endothelial growth factors and hepatocyte growth factors, which contribute to the regeneration of damaged organs compared with BMSC [16]. Thus, ADSCs are expected to serve as an excellent material in regenerative treatments of patients with rapidly progressive and severe DM using the secretion mechanism for cell growth factors, immune regulators, and tissue-regenerating factors [17].

ADSC transplantation efficiency is an important factor for successful ADSC transplantation, wherein the aim is to achieve regenerative treatment effects using the secretion mechanism for cell growth factors, immune regulators, and tissue-regenerating factors. However, it was not revealed whether the transplanted autologous ADSCs derived from patients with DM may be engrafted similarly to those derived from healthy persons. To address this limitation, this study clarified the difference in transplantation efficiency between ADSCs derived from healthy individuals (normal ADSCs) and ADSCs derived from patients with type II DM (T2DM ADSCs).

## 2. Results

### 2.1. Transplantation Efficiency of T2DM ADSCs and Normal ADSCs

**Figure 1:**
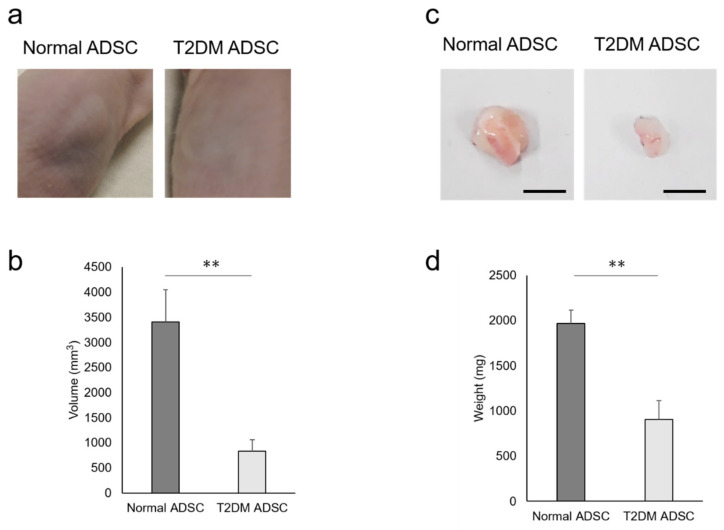
Evaluation of the transplantation efficiencies of ADSCs derived from patients with type II diabetes mellitus. The normal ADSCs and T2DM ADSCs were transplanted subcutaneously into the backs of immunodeficient mice. (**a**) The images of the appearances of the transplant sites in immunodeficient mice 2 weeks after the transplantation are shown. (**b**) Two weeks after the transplantation, the volumes (mm^3^) of the transplantation sites were measured as mean ± SE. The number of samples was *n* = 6. The volume was evaluated three times independently. The unpaired *t*-test was used to test for statistical significance. ** *p* value = 0.006. (**c**) The images of the appearances of the skin grafts removed 3 weeks after the transplantation are shown. The scale bar indicates 10 mm. (**d**) Three weeks after the transplantation, the skin grafts were removed, and their weights (mg) were taken as mean ± SE. The number of samples was *n* = 6. The weight was evaluated three times independently. The unpaired *t*-test was used to test for statistical significance. ** *p* value = 0.0037.

To clarify the difference in transplantation efficiency between T2DM and normal ADSCs, both were mixed with Matrigel and growth factors and then transplanted into the subcutaneous tissues of the backs of immunodeficient mice (strain name BALB/cAJcl-nu/nu). After two weeks, the sizes of transplant sites in the normal ADSC transplantation group were larger than those in the T2DM ADSC group (Figure 1a). The average volume of the transplant sites was 3407 ± 637 mm^3^ in the normal ADSC group, equivalent to four times that in the T2DM ADSC group, 834 ± 226 mm^3^ (Figure 1b). The skin grafts removed 3 weeks after the transplantation were larger in the normal ADSC group than in the T2DM ADSC group (Figure 1c). The average weight of the skin grafts was 1967 ± 151 mg in the normal ADSC group, which is equivalent to approximately 2.2 times that of the T2DM ADSC group, 903 ± 208 mg (Figure 1d). Thus, the volumes and weights of the skin grafts were significantly lower in the T2DM ADSC group than in the normal ADSC group, suggesting that the former might have lower transplantation efficiency.

### 2.2. Proliferation Potency and Stemness of T2DM ADSCs and Normal ADSCs

**Figure 2:**
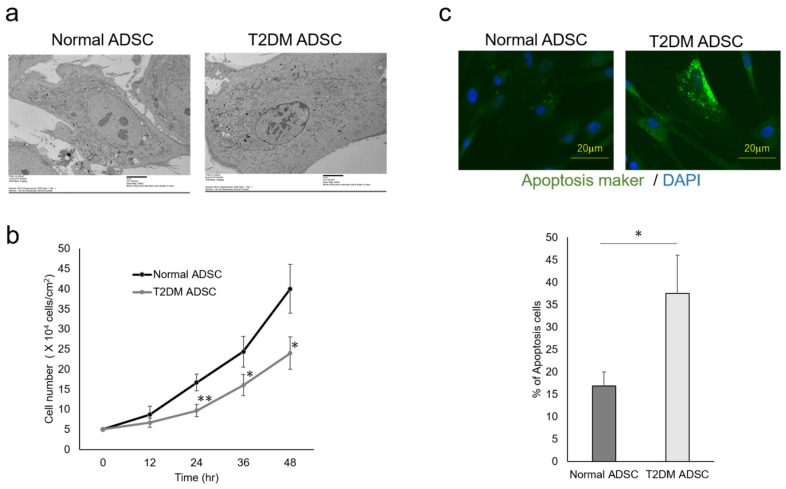
Evaluation of the cell characteristics of ADSCs derived from patients with type II diabetes mellitus. The cell characteristics of normal ADSCs and T2DM ADSCs were evaluated. (**a**) The electron micrographs of the normal ADSC and T2DM ADSC cellular structures, and the difference between the two images was observed. The scale bar indicates 6 μm. (**b**) The difference in cell growth rate in a 12 h time-course variation of cell count (cells/cm^2^) is shown. ** *p* vales = 0.0093 (24 h), * *p* vales = 0.0354 (36 h), * *p* vales = 0.019 (48 h). (**c**) The difference in cell death in terms of positive cell rate is shown. The apoptotic marker-positive cells were detected by staining with a fluorescent dye, FITC, using an apoptotic detection kit, and the positive cell rates are shown as the mean ± SE. The number of samples was *n* = 3. The percentage of apoptosis maker-positive cells was evaluated three times independently. The unpaired *t*-test was used to test for statistical significance. * *p* value = 0.0173. The scale bar indicates 20 μm.

**Figure 3:**
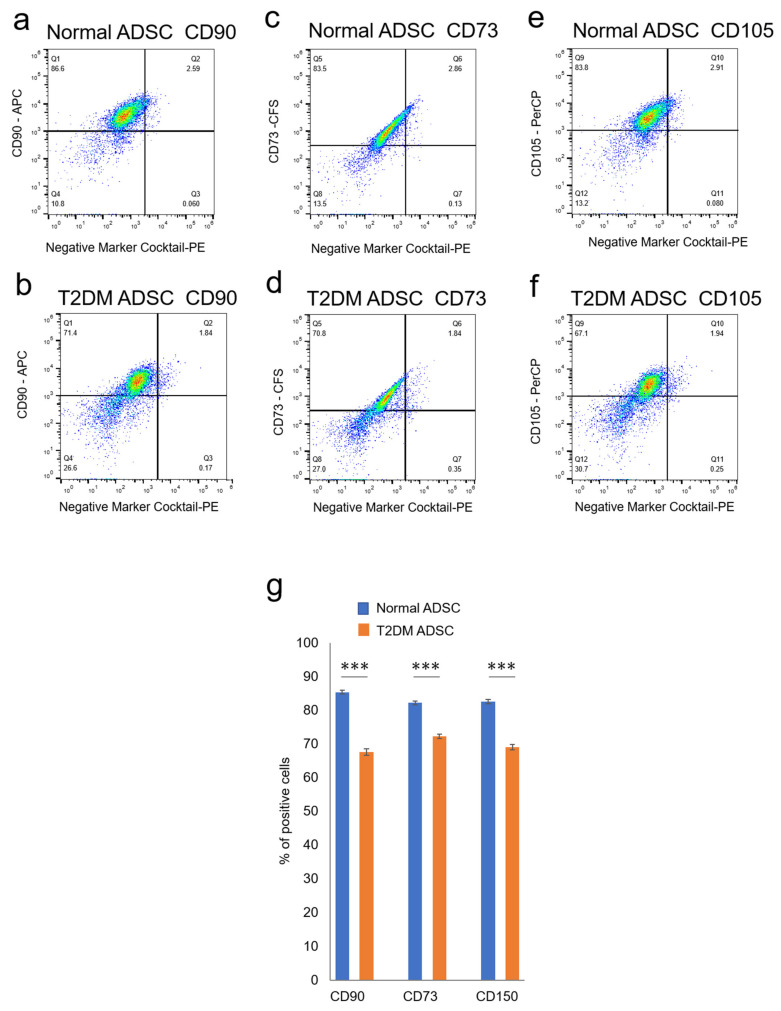
Evaluation of the stemness of ADSCs derived from patients with type II diabetes mellitus. The variation in stem cell rates of normal and T2DM ADSCs was evaluated by flowcytometry. (**a**) The distribution of the stem cell marker, CD90, in the normal ADSCs is shown. (**b**) The distribution of the stem cell marker, CD90, in the T2DM ADSCs is shown. (**c**) The distribution of the stem cell marker, CD73, in the normal ADSCs is shown. (**d**) The distribution of the stem cell marker, CD73, in the T2DM ADSCs is shown. (**e**) The distribution of the stem cell marker, CD105, in the normal ADSCs is shown. (**f**) The distribution of the stem cell marker, CD105, in the T2DM ADSCs is shown. The stem cell marker-positive cell rates were calculated. (**g**) The stem cell marker-positive cell rates in terms of mean ± SE are shown. The number of samples was *n* = 3. The percentage of CD maker-positive cells was evaluated three times independently. The unpaired *t*-test was used to test for statistical significance. For CD90, *** *p* = 0.0002. For CD73, *** *p* = 0.0006. For CD105, *** *p* = 0.0004.

The differences in cell characteristics were evaluated between the normal and T2DM ADSCs. The observation of the differences in cell structure under the TEM demonstrated that T2DM ADSCs were more hypertrophied than normal ADSCs (Figure 2a). Furthermore, it was clarified that the cell growth rate of T2DM ADSCs was lower than that of normal ADSCs (Figure 2b). Moreover, the stem cell rates of normal and T2DM ADSCs were evaluated by flow cytometry. The result showed that the rate of stem cell marker-positive T2DM ADSCs was significantly lower than that of normal ADSCs (Figure 3). The difference in apoptotic cell death was examined. The result showed that the rate of apoptotic marker-positive T2DM ADSCs was significantly higher than that of normal ADSCs (Figure 2c). Thus, it was suggested that T2DM ADSCs have lower proliferating potency and stemness than normal ADSCs, leading to a tendency to induce apoptotic cell death.

### 2.3. Expression of Fox Signaling Pathway-Related Genes by T2DM ADSCs and Normal ADSCs

**Figure 4:**
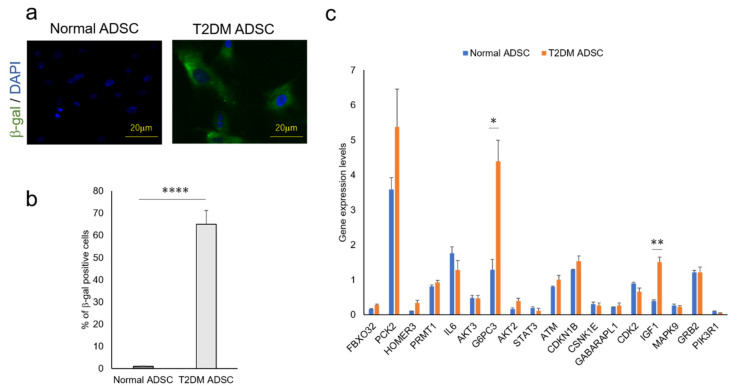
Quantitative analysis of cell aging of ADSCs derived from patients with type II diabetes mellitus and the genes related to the FoxO signaling pathway. The difference in cell aging between the normal and T2DM ADSCs and the expression levels of the genes related to the FoxO signaling pathway were quantified. (**a**) The images of aged cells labeled with green fluorescent dye using a cell aging marker, β-gal, are shown. The aging marker-positive cells were counted. (**b**) The positive cell rates in terms of mean ± SE are shown. The number of samples was *n* = 3. The percentage of β-gal positive cells was evaluated three times independently. The unpaired *t*-test was used to test for statistical significance. **** *p* value < 0.0001. (**c**) The genes related to the FoxO signaling pathway in normal and T2DM ADSCs were quantified using quantitative PCR. The quantified genes in terms of mean ± SE. The number of samples was *n* = 3. The gene expression level was evaluated three times independently. The unpaired *t*-test was used to test for statistical significance. For *G6PC3*, * *p* = 0.0196. For *IGF1*, ** *p* = 0.0035.

The factors that cause the reduction in the T2DM ADSC transplantation efficiency and control the differences in cell characteristics are unclear. The genes involved in these mechanisms were revealed in a cyclopedic mRNA analysis using a next-generation sequencer. The differentially expressed genes of normal and T2DM ADSCs were detected by a next-generation sequencer. The distribution of the gene expression data (MA-plot) shown in the Supplementary Figure S1. The genes with expression variation ≥4 times higher than normal ADSCs were then extracted from T2DM ADSCs by KEGG PATHWAY analysis using DAVID. The complete list of differently expressed genes are listed in the Supplementary Table S1. The raw results were uploaded to the NCBI publicly available database (BioSample accessions SAMN19651932, SAMN19651933). Eight pathways, namely, acute myeloid leukemia, Epstein–Barr virus infection, protein processing in endoplasmic reticulum, prostate cancer, ribosome, metabolic, FoxO signaling, and glycerophospholipid metabolism, were identified (Table 1). Furthermore, it was clarified that T2DM ADSCs have a significantly higher rate of cells positive for senescence-associated-beta-galactosidase than normal ADSCs, demonstrating that cell aging appears to be induced in T2DM ADSCs (Figure 4b).

In light of the above results, we focused on the FoxO signaling pathway, which was reported to be involved in cell aging. The next-generation sequencer identified 18 genes related to the FoxO signaling pathway (Table 2). The quantitative PCR result revealed that the expression levels of glucose-6-phosphatase 3 (*G6PC3*) and insulin-like growth factor (*IGF1*) genes were significantly higher in T2DM ADSCs than in normal ADSCs (Figure 4c).

### 2.4. Involvement of the G6PC3 and IGF1 Genes Related to the FoxO Signaling Pathway in the Reduction in T2DM ADSC’s Transplantation Efficiency

**Figure 5:**
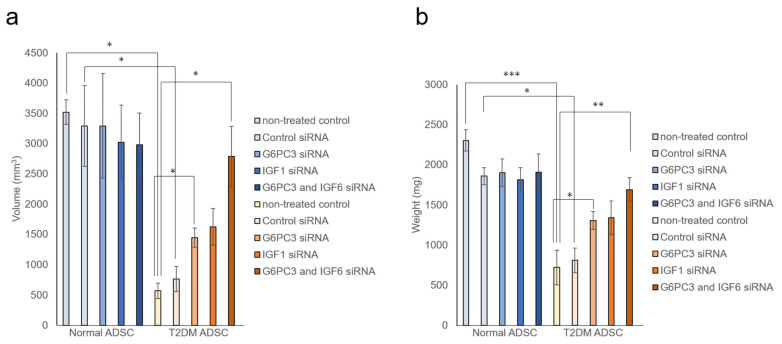
Evaluation of the improvement in transplantation efficiency by knocking down the *G6PC3* and *IGF1* genes of ADSCs derived from patients with type II diabetes mellitus. It was evaluated whether knockdown the *G6PC3* and *IGF1* genes might have an effect on normal ADSC and T2DM ADSC transplantation efficiency. The normal ADSCs and T2DM ADSCs with siRNA mediated knockdown of *G6PC3* and *IGF1*C were transplanted into the backs of immunodeficient mice. (**a**) The volumes (mm^3^) of the transplantation sites were measured 2 weeks after the transplantation. The measured volumes in terms of mean ± SE are shown. The number of samples was *n* = 3. The volume was evaluated three times independently. The unpaired *t*-test was used to test for statistical significance. * *p* = 0.0326 (Normal ADSC non-treated cells vs T2DM non-treated cells). * *p* = 0.0102 (Normal ADSC control siRNA vs T2DM control siRNA). * *p* = 0.0234 (T2DM ADSC non-treated cells vs T2DM *G6PC3* and IGF6 siRNA). * *p* = 0.0169 (T2DM ADSC non-treated cells vs T2DM G6PC siRNA). * *p* = 0.0380 (T2DM ADSC control siRNA vs T2DM *G6PC3* and IGF6 siRNA). (**b**) The skin grafts were removed and weighed (mg) 3 weeks after the transplantation. The measured weights in terms of mean ± SE are shown. The number of samples was *n* = 3. The weight was evaluated three times independently. The unpaired *t*-test was used to test for statistical significance. *** *p* = 0.0004 (Normal ADSC non-treated cells vs T2DM non-treated cells). * *p* = 0.0102 (Normal ADSC control siRNA vs T2DM control siRNA). ** *p* = 0.0095 (T2DM ADSC non-treated cells vs T2DM *G6PC3* and IGF6 siRNA). * *p* = 0.0267 (T2DM ADSC non-treated cells vs T2DM G6PC siRNA). ** *p* = 0.0280 (T2DM ADSC control siRNA vs T2DM *G6PC3* and IGF6 siRNA).

**Figure 6:**
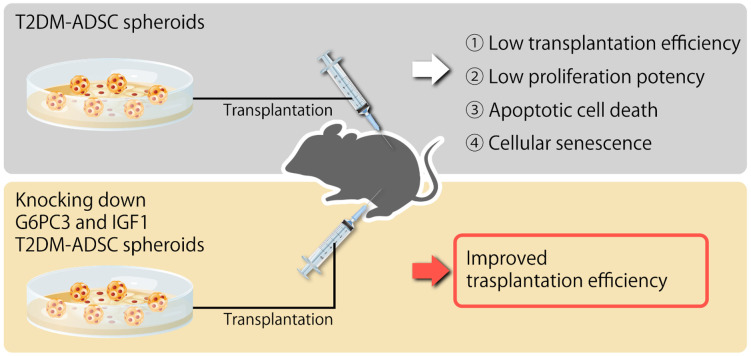
Summary diagram of this study.

It was evaluated whether the *G6PC3* and *IGF1* genes might be involved in reducing the transplantation efficiency of T2DM ADSC. The siRNA of *G6PC3* and *IGF1* genes were introduced into normal and T2DM ADSCs and confirmed to reduce the target gene expression levels by ≥90%. The normal ADSCs and T2DM ADSCs with knockdown of *G6PC3* and *IGF1* were transplanted subcutaneously into the backs of immunodeficient mice (strain name BALB/cAJcl-nu/nu). In the T2DM ADSC group, the average volume of the transplant sites 2 weeks after the transplantation increased to 1445 ± 159 mm^3^ in the *G6PC3* siRNA subgroup and 1624 ± 304 mm^3^ in the *IGF1* siRNA subgroup, and further increased to 2785 ± 499 mm^3^ in the *G6PC3* and *IGF1* siRNA combined subgroup compared with 766 ± 207 mm^3^ in the control siRNA subgroup (Figure 5a). The average weight of the skin grafts removed 3 weeks after the transplantation increased to 1305 ± 112 mg in the *G6PC3* siRNA subgroup and 1338 ± 210 mg in the *IGF1* siRNA subgroup, and further increased to 1692 ± 148 mg in the *G6PC3* and *IGF1* siRNA combined subgroup compared with 810 ± 154 mg in the control siRNA subgroup (Figure 5b). Thus, it was clarified that the G6PC and *IGF1* genes related to the FoxO signaling pathway might reduce the transplantation efficiency of T2DM ADSC (Figure 6).

## 3. Discussion

In rapidly progressive and severe DM cases accompanied by complications, autologous stem cell transplantation, one of the regenerative treatments, is preferable. However, it was unknown whether it is as effective as in healthy individuals that undergo an autonomous transplantation using the stem cells of DM patients. This study revealed the difference in transplantation efficiency between the normal ADSCs and T2DM ADSCs.

In summary, this study clarified that the volumes and weights of the skin grafts were significantly lower in the transplantation of T2DM ADSCs than those of the normal ADSCs, suggesting that T2DM ADSCs have lower transplantation efficiencies (Figure 1). Thus, the differences in cell characteristics between T2DM and normal ADSCs were examined. The result showed that T2DM ADSCs had lower proliferation potency and stemness than normal ADSCs, leading to a tendency to induce apoptotic cell death (Figure 2 and Figure 3). To elucidate the reason behind this, we conducted a cyclopedic mRNA analysis using a next-generation sequencer. We identified the genes involved in the lower transplantation efficiencies of T2DM ADSCs and the differences in cell characteristics. KEGG pathway analysis was conducted on extracted genes using the DAVID. The analysis identified eight pathways, and we focused on the FoxO signaling pathway (Figure 4b and Table 1), which was reported to be involved in cell aging. The next-generation sequencer identified 18 FoxO signaling pathway-related genes. The quantitative PCR results revealed that *G6PC3* and IGF1 genes’ expression levels were significantly higher in T2DM ADSCs than in normal ADSCs (Figure 4c and Table 2). The *G6PC3* and IGF1 genes related to the FoxO signaling pathway were involved in the reduction in T2DM ADSC transplantation efficiency (Figure 5). To our knowledge, this is the first report to present the differences in the transplantation efficiencies between ADSCs derived from healthy individuals and patients with type II DM and the genes responsible for these differences (Figure 6).

The FoxO signaling pathway highlighted in this study is an important pathway to control the cell cycle under intense starvation stress [18] and induce cell death [19] and cell aging [20]. FoxO is a transcription factor controlled at the downstream of the insulin receptor and activated mainly in response to starvation stress [21,22]. The *IGF1* gene identified in this study is a FoxO signaling pathway-related gene, and it is referred to as somatomedin C [22]. Somatomedin C is a hormone with an insulin-like molecular structure [22]. This study revealed that reducing the *IGF1* expression level might improve the T2DM ADSC transplantation efficiencies. *IGF1* is a single-strand polypeptide with 7649 Daltons composed of 70 amino acids. It is encoded by the human *IGF1* gene and has three disulfide bonds in the molecule [23]. *IGF1* is primarily produced in the liver as an endocrine hormone and produced in multiple tissues as a paracrine or autocrine hormone [24]. The *IGF1* production is stimulated by the growth hormones [24]. The production of *IGF1* is inhibited by malnutrition, insusceptibility to the growth hormone, and growth hormone receptor deficiency [25]. *IGF1* primarily binds to an *IGF1* receptor and receptor tyrosine kinases of the insulin receptor [25]. *IGF1* activates the insulin signaling pathway and PI3K-Akt signaling pathway, regulates DNA synthesis, and controls the cell death, cycle, and metabolism [26]. This study reported that knocking down the *IGF1* gene in T2DM ADSCs might improve the transplantation efficiencies and revealed a new role of the *IGF1* gene in stem cells, which has not been reported to date.

The *G6PC3* gene related to the FoxO signaling pathway identified in this study referred to as glucose-6-phosphatase-β encodes the catalytic subunit of the glucose-6-phosphatase (G6Pase) [27]. G6Pase is localized in the endoplasmic reticulum and catalyzes the hydrolysis of glucose-6-phosphoric acid in the glycolytic and glycogenolytic pathways [28]. This study reported that knocking down the *G6PC3* gene in the T2DM ADSCs might improve transplantation efficiencies and revealed the new role of the *G6PC3* gene in stem cells, which has not been reported to date.

Several limitations of this study should be considered. First, this study found that knockdown of both G6PC and *IGF1* improved transplant resistance of stem cells derived from patients with type II diabetes. The source of these stem cells was human adipose tissue, but this study uses immunodeficient mice as recipients. Therefore, it has not been proven the efficacy of this genetic treatment in allograft transplantation using stem cells derived from type II diabetic patients. As a future issue, it is hoped that safety of this genetic approach will be confirmed and clinical trials will be conducted. Next, in this study, we focused on the FoxO signal pathway by next-generation sequencing and found *G6PC3* and *IGF1*. It is still unclear by what molecular mechanism *G6PC3* and *IGF1* cause treatment resistance of stem cells derived from type 2 diabetic patients, and detailed analysis will be conducted in the future.

The current experimental findings are scientifically and clinically important. Mesenchymal stem cells are an excellent cell material for regenerative therapy, but the transplant ability of stem cells derived from patients with underlying diseases such as diabetes has not been clarified. Additionally, ADMSCs can be isolated with high yield by relatively low-invasive procedure. Recent studies are showing that co-transplantation with ADMSCs can increase islet survival and function, which rescues from islets loss in regenerative therapy [29]. This study revealed that stem cells from patients with type 2 diabetes are resistant to transplantation. It also revealed that knocking down of both *G6PC3* and *IGF1* improves transplant resistance. The results of this study revealed specific ways to improve the success rate of transplantation when diabetics perform regenerative therapy using autologous stem cells.

The results of this study suggest that the use of T2DM ADSCs in regenerative treatments helps to reduce regeneration efficiencies, and the reduction in the expression levels of *G6PC3* and *IGF1* genes has the potential to improve transplantation efficiencies. These results will help to improve the quality control of stem cells to enhance their transplantation efficiencies, leading to the spread of regenerative treatments using autologous stem cells.

## 4. Materials and Methods

### 4.1. Materials and Correspondence

Type II diabetes mellitus adipose-derived stem cells (T2DM ADSC) (Lonza, Greenwood, SC, USA);

Normal adipose-derived stem cells (Normal ADSC) (Lonza, Greenwood, SC, USA);

ADSC Apidose-Derived Stem Cells Growth Medium BulletKit™ (Lonza, Greenwood, SC, USA);

0.05% Trypsin EDTA (Gibco, Co Dublin, Ireland);

10 cm^2^ Culture dish (100 mm × 20 mm Style dish) (Corning, Glendale, AZ, USA);

Matrigel^®^ Matrix For Organoid Culture Phenol Red Free (Corning, Glendale, AZ, USA);

Matrigel^®^ Matrix Basement Membrane HC Growth Factor Reduced (Corning, Glendale, AZ, USA);

Mice BALB/cAJcl-nu/nu (BALB/cAJcl-Foxn1nu) (CLEA Japan, Inc., Tokyo, Japan);

60 × 15 mm^2^ Tissue culture dish (FALCON/Corning, Glendale, AZ, USA);

Glutaraldehyde (EM grade, Electron Microscopy Science, Hatfield, PA, USA);

Osmium tetra-oxide (Crystal, Heraeus Chemicals South Africa, Boksburg, South Africa);

Ethanol (Wako, Osaka, Japan);

Toluidine bleu (Wako, Osaka, Japan);

Grid: Cu 200 mesh EM fine grid (Nisshinn EM, Tokyo, Japan);

Epoxy resin (TAAB Laboratories, Tokyo, Japan);

In situ Apoptosis Detection Kit (TAKARA, Shiga, Japan);

ProLong™ Gold Antifade Mountant with DAPI (Invitrogen, Tokyo, Japan);

Human Mesenchymal Stem Cell Verification Multi-Color Flow Cytometry Kit (R&D Systems, Minneapolis, MN, USA);

RNeasy Mini Kit (QIAGEN, Hilden, Germany); 

Cellular Senescence Detection Kit—SPiDER-bGal (DOJINDO, Kumamoto, Japan);

PrimeScript^®^ RT reagent Kit (Perfect Real Time) (TAKARA, Shiga, Japan);

SYBR^®^ Premix Ex Taq^TM^ II (Tli RNaseH Plus) (TAKARA, Shiga, Japan);

TAKARA Perfect Real Time Primer (TAKARA, Shiga, Japan);

Silencer^®^ Select Pre-designed (Inventoried) siRNA (Thermo Fisher Scientific, Waltham, MA, USA);

Eight-well cell culture slides (SPL Life Sciences, Gyeonggi-do, Korea);

MTT Cell Proliferation and Cytotoxicity Assay Kit (Boster, Pleasanton, CA, USA).

### 4.2. Normal ADSCs and T2DM ADSCs Culture

Normal ADSCs and T2DM ADSCs were purchased from Lonza. The ADSCs were seeded on 10 cm^2^ Petri dishes filled with 15 mL of medium containing cell growth factors at a density of ~5000 cells/cm^2^. The ADSCs were cultured in an incubator at 5% CO concentration and 37 °C. After proliferating to ~70–80% of the culture container area, the ADSCs were seeded on other Petri dishes at a density of ~5000 cells/cm^2^ to maintain the culture. The cells were stripped using 3 mL trypsin/EDTA solution per Petri dish.

### 4.3. ADSC Transplantation into Immunodeficient Mice and Measurement of Volumes and Weights of the Transplant Sites

Normal ADSCs and T2DM ADSCs were cultured to form spheroids 1 week before the transplantation by adding cell growth factors supplied with the Matrigel matrix for spheroid culture and the selective medium. The stem cell spheroids were mixed with the Matrigel matrix at a ratio of 1:1. Note that ~300 μL of the mixture containing 10^7^ ADSCs was filled in a syringe on ice and transplanted into the subcutaneous tissues of the backs of immunodeficient mice (strain name, BALB/cAJcl-*nu*/*nu*). Two weeks after normal ADSC and T2DM ADSC transplantation, the width, depth, and height of the transplant sites were measured using a caliper to calculate the volume (mm^3^). Three weeks after the transplantation, the skin grafts were removed to measure their weights (mg) using a precision electronic balance.

### 4.4. Evaluation of ADSC Structures Using a Transmission Electron Microscope

The normal ADSCs and T2DM ADSCs with a density of ~5000 cells/cm^2^ were seeded on 3.5 cm^2^ Petri dishes filled with 5 mL of specific medium containing the cell growth factors. To adjust the samples for observation under a transmission electron microscope (TEM), the seeded cells were prefixed at 4 °C overnight using phosphate buffer saline (PBS) containing 2% glutaraldehyde. The samples were washed with PBS and postfixed at 4 °C for 2 h using an Osmium 2% aqueous solution. The samples were dehydrated with ethanol concentration elevated from 30% to 100% for 15 min for each stage. The samples were embedded in epoxy resin at 60 °C for 48 h, cut into 80–90 nm-sized superthin sections using an ultramicrotome, and loaded onto a Cu 200 mesh. The loaded samples were stained with 2% uranium acetate aqueous solution for 15 min, followed by lead stain for 5 min, and observed at 100 kV using TEM H-7600.

### 4.5. Evaluation of the Cell Counts and Death of ADSCs

To evaluate the cell count of ADSCs, normal ADSCs and T2DM ADSCs with a density of ~5000 cells/cm^2^ were seeded on 24-well plates filled with a medium containing cell growth factor. The cell count was taken every 12 h by a fully automatic cell counter, TC20.

To evaluate the cell death of ADSCs, an in situ apoptosis detection kit was used. The TUNEL method was used to detect apoptosis. The free 3′-OH terminal of fragmented DNA of apoptotic cells by the TUNEL method is highly efficiently and specifically labeled with fluorescein-dUTP using terminal transferase (TdT), and green fluorescence is detected by fluorescence microscopy. Detection of apoptotic cells by the TUNEL method was performed with a fluorescence microscope. Apoptosis-positive cells by the TUNEL method were detected and measured by the fluorescence intensity using KEYENCE fluorescence observation software. The normal ADSCs and T2DM ADSCs with a density of ~5000 cells/cm^2^ were seeded on 8-well chamber slides filled with a medium containing the cell growth factors. Then, 48 h after cell seeding, the cells were fixed with 4% paraformaldehyde at room temperature for 20 min. Moreover, ~100 μL of permeabilization buffer was added to enhance the permeability of the liquid enzymatic reaction mixture. The reaction was induced on ice for 5 min; the cells were washed with PBS. Adjustment liquids, TdT enzyme, and labeling-safe buffer were added on the slides to induce a reaction in a wet box at 37 °C for 90 min. After the completion of the reaction, the cells were washed with PBS to stop the reaction. Anti-FITC HRP conjugate was reacted with the cells at 37 °C for 30 min; after the completion of the reaction, the cells were washed with PBS to stop the reaction. The samples were mounted using the mounting medium with DAPI (4′,6-Diamidino-2-phenylindole dihydrochloride). The apoptosis marker and DAPI were observed under the inverted fluorescence phase-contrast microscope, BZ-X700.

### 4.6. Evaluation of the ADSC-Specific Marker-Positive Rate Using Flow Cytometry

The stemness of normal ADSCs and T2DM ADSCs was evaluated using the Human Mesenchymal Stem Cell Verification Multi-Color Flow Cytometry Kit. For ADSC-specific-positive markers, CD90 antibody labeled with APC fluorescence dye, CD73 antibody labeled with CFS fluorescence dye, and CD 105 antibody labeled with PerCP fluorescence dye were used. For ADSC-specific-negative markers, PE-labeled CD45, CD34, CD11b, CD79A, and HLA-DR were used. An isotype control was used as a negative control for each antibody.

The normal ADSCs and T2DM ADSCs with a density of ~5000 cells/cm^2^ were seeded on six-well plates filled with a medium containing cell growth factors. Then, 24 h after cell seeding, the cells were collected, washed with staining buffer, and centrifuged at 300 × *g* for 5 min. The cell count was adjusted to 1 × 10^6^ cells/mL, and the cells were blocked with Fc receptor blocking reagents. For positive markers, 10 µL of each CD90, CD73, and CD105 antibodies and 10 µL of negative marker cocktail (containing CD45, CD34, CD11b, CD79A, and HLA-DR antibodies) were added to induce the reaction at room temperature for 45 min. After staining, the cells were washed with staining buffer, and the cell pellets were resuspended in 400 µL of staining buffer. The samples were analyzed using a flow cytometer, SA3800. The obtained analytical data were analyzed using FLOWJO, and the positive cell rate was calculated.

### 4.7. Gene Expression Variation Analysis Using a Next-Generation Sequencer

The normal ADSCs and T2DM ADSCs with a density of ~5000 cells/cm^2^ were seeded on 10 cm^2^ Petri dishes filled with 15 mL of medium containing cell growth factors. On day 8 of culture, the cells were collected by a scraper, washed with 1 × PBS, and adjusted to 6 × 10^5^ cells. Total RNA of each sample was extracted using RNeasy Mini Kit (QIAGEN). As a result of measuring the concentration of the obtained Total RNA with Nano Drop ONE, the total yield of Total RNA was: Normal-ADSC, 8.8 µg; Type I-ADSC, 5.2 µg; and Type II-ADSC, 8.1 µg. In order to confirm the purity of Total RNA, RIN (RNA Integrity Number) was measured by Bioanalyzer (Agilent, Santa Clara, CA, USA). RIN is a score of RNA quality from 1 to 10 and uses a RIN value of 7 or higher for next-generation sequence analysis. As a result of the measurement, the RIN values were: Normal-ADSC, 9.8; Type I-ADSC, 10; Type II-ADSC, 9.9. These could be used for next-generation sequence analysis. Using this Total RNA, we proceeded to the extraction process of poly (A) RNA. Using 100 ng of Total RNA, poly (A) RNA was extracted and fragmented using NEBNext Poly (A) mRNA Magnetic Isolation Module (NEB, Ipswich, MA, USA) and NEBNext Ultra II RNA Library Prep Kit for Illumina (NEB). The protocol followed the NEBNext Ultra II RNA Library Prep Kit for Illumina Instruction Manual. Fragmentation was performed at 94 °C for 15 min by adding NEBNext First Strand Synthesis Reaction Buffer and NEBNext Random Primers from NEBNext Ultra II RNA Library Prep Kit for Illumina (NEB). Reverse transcription reaction was performed on fragmented poly (A) RNA using NEBNext First Strand Synthesis Enzyme Mix of NEBNext Ultra II RNA Library Prep Kit for Illumina (NEB) to prepare cDNA, and then NEBNext Adapter (NEB) was used. An adapter was also added. The prepared cDNA was amplified by PCR to prepare a library. At this time, NEBNext Multiplex Oligos for Illumina was used to add 18, 19, and 20 barcode sequences to identify the sample. As a result of measuring the concentration of the prepared library using Qubit (Thermo Fisher Scientific), the concentration was: Normal-ADSC, 7.28 ng/µL; Type I-ADSC, 4.74 ng/µL; Type II-ADSC, 7.42 ng/µL. It also performed a Bioanalyzer analysis to confirm the library length distribution. As a result of the analysis, a library of the desired length was created, and sufficient concentration and purity were confirmed, so we proceeded to next-generation sequence analysis. The Illumina NextSeq analysis used fragment analysis to analyze the cDNA region 75 bp and the barcode sequence. CLC Genomics Workbench 12.0.3 was used for RNA-Seq data analysis. After importing the fastq file output from Illumina NextSeq into GWB, the leads were trimmed with GWB’s Trim reads 2.3 tool. The parameter settings were the default settings, except for the 3′end which was deleted by 1 bp. For expression analysis, the trimmed reads were mapped to reference sequences and tag counts were performed using the GWB RNA-Seq Analysis 2.18 tool. For the reference sequence and genome annotation, we used Homo sapience GRCh37 downloaded from Ensemble by the function of GWB.

Function analysis used The Database for Annotation, Visualization and Integrated Discovery (DAVID) v6.8. The genes that showed 4-fold or more expression variation in the T2DM group compared with Normal were analyzed with DAVID software, and the corresponding KEGG pathway was identified.

### 4.8. Evaluation of ADSCs Cellular Senescence

To evaluate cellular senescence, the Cellular Senescence Detection Kit—SPiDER-βGal—was used. The normal ADSCs and T2DM ADSCs with a density of ~5000 cells/cm^2^ were seeded on 8-well chamber slides filled with a medium containing cell growth factors. Then, 48 h after cell seeding, the cells were washed with HBSS. The prepared Bafilomycin A1 working solution was added, and the cells were incubated for culture at 5% CO_2_ concentration and 37 °C for 1 h. The prepared SPiDER-βGal working solution was added, and the cells were left to stand in an incubator for culture for 30 min. The samples were mounted using the mounting medium with DAPI. The cell aging marker and DAPI were observed under the inverted fluorescence phase-contrast microscope, BZ-X700.

### 4.9. Determination of Gene Expression Levels by the Quantitative PCR Method

The normal ADSCs and T2DM ADSCs with a density of ~5000 cells/cm^2^ were seeded on 10 cm^2^ Petri dishes filled with 15 mL of medium containing growth factors. The cells were collected with a cell scraper on day 8 of culture, washed with PBS, and adjusted to 6 × 10^5^ cells. The messenger RNA (mRNA) was extracted using the RNeasy Mini Kit. A template cDNA was obtained from the extracted mRNA through reversal transcription using the PrimeScriptR RT reagent Kit (Perfect Real Time).

In quantitative PCR, SYBR^®^ Premix Ex Taq™ II (Tli RNaseH Plus) was used. Primers were purchased from the TAKARA Perfect Real Time Primer support system. Using the template cDNA, PCR amplification was conducted using SYBR Premix Ex TaqTM II with forward and reverse primers on a quantitative PCR device, StepOne-Plus-01. First, denaturing was performed at 95 °C for 30 s. Then, a cycle of amplification at 95 °C for 5 s and 60 °C for 60 s was repeated 40 times. The expression levels of the target genes were calculated using a calibration curve, and the gene expression ratios were determined using GAPDH as the internal standard. Primer information is provided in Supplementary Table S2.

### 4.10. Evaluation of the Transplantation Effects by Knocking down the G6PC3 and IGF1 Genes

In an experiment of knocking down the *G6PC3* and *IGF1* genes, Silencer^®^ Select Pre-designed (Inventoried) siRNA was used. For the siRNA sequences of *G6PC3*, (5′)-GGA UCU UUC UUG GUC CAU CAG CCU A-(3′) and (5′)-UAG GCU GAU GGA CCA AGA AAG AUC C-(3′) were used. For the siRNA sequences of *IGF1*, (5′)-CGA GUU ACC UGU UAA ACU U-(3′) and (5′)-AAG UUU AAC AGG UAA CUC G-(3′) were used. The normal ADSCs and T2DM ADSCs with a density of ~5000 cells/cm^2^ were seeded on 10 cm^2^ Petri dishes filled with 15 mL of medium containing growth factors. Then, 24 h after cell seeding, the siRNA for *G6PC3* and *IGF1* genes was transfected using an electroporation device, NEPA21-S. The gene knocking down efficiency was determined using a quantitative PCR device, StepOne-Plus-01; the result showed that the expression levels of the target genes were reduced by ≥90%. The normal ADSCs and T2DM ADSCs with knocked down target genes were cultured to form spheroids 1 week before the transplantation by adding cell growth factors supplied with the Matrigel matrix for spheroid culture and the selective medium. The stem cell spheroids were mixed with the Matrigel matrix (for transplantation) at a ratio of 1:1. The mixture containing 10^7^ ADSCs was filled in a syringe on ice and transplanted into the subcutaneous tissues of the backs of immunodeficient mice. Two weeks after the transplantation, the width, depth, and height of the transplant sites were measured using a caliper to calculate the volume (mm^3^). Three weeks after normal ADSC and T2DM ADSC transplantation, the skin grafts were removed to measure their weights (mg) using a precision electronic balance.

## Figures and Tables

**Table 1:** Listing of KEGG pathways of differentially expressed genes in ADSCs derived from patients with type II diabetes mellitus using a next-generation sequencer.

KEGG PATHWAY	Genes	Cover %	*p*-Value	Benjamini
Acute myeloid leukemia	9	0.5	9.60 × 10^−2^	6.00 × 10^−1^
Epstein-Barr virus infection	16	1	8.80 × 10^−2^	5.70 × 10^−1^
Protein processing in endoplasmic reticulum	21	1.3	7.50 × 10^−2^	5.30 × 10^−1^
Prostate cancer	13	0.8	6.50 × 10^−2^	4.90 × 10^−1^
Ribosome	18	1.1	6.40 × 10^−2^	5.00 × 10^−1^
Metabolic pathways	118	7.1	6.10 × 10^−2^	4.90 × 10^−1^
FoxO signaling pathway	18	1.1	5.70 × 10^−2^	4.80 × 10^−1^
Glycerophospholipid metabolism	14	0.8	5.50 × 10^−2^	4.80 × 10^−1^

**Table 2:** Listing of differentially expressed genes related to the FoxO signaling pathway in ADSCs derived from patients with type II diabetes mellitus using a next-generation sequencer.

Gene Name	T2DM/Normal Ratio	Transcript Length	Exons	Gene ID
*FBXO32*	4.607968001	735	3	ENSG00000156804
*PCK2*	4.607968001	525	3	ENSG00000100889
*HOMER3*	4.607968001	770	4	ENSG00000051128
*PRMT1*	5.160924161	766	7	ENSG00000126457
*IL6*	5.759960001	985	4	ENSG00000136244
*AKT3*	5.792874059	3588	13	ENSG00000117020
*G6PC3*	5.990358401	805	6	ENSG00000141349
*AKT2*	5.990358401	954	3	ENSG00000105221
*STAT3*	6.451155202	826	3	ENSG00000168610
*ATM*	7.065550935	4841	27	ENSG00000149311
*CDKN1B*	8.063944002	897	2	ENSG00000111276
*CSNK1E*	8.294342402	2321	15	ENSG00000213923
*GABARAPL1*	8.820967316	2321	6	ENSG00000139112
*CDK2*	9.215936002	2598	6	ENSG00000123374
*IGF1*	9.215936002	7370	5	ENSG00000017427
*MAPK9*	11.9807168	657	5	ENSG00000050748
*GRB2*	13.3631072	687	4	ENSG00000177885
*PIK3R1*	31.33418241	6435	15	ENSG00000145675

## Data Availability

Not applicable.

## Supplementary Materials

The following are available online at https://www.mdpi.com/article/10.3390/ijms22126595/s1.
