# Supplementary material for: The Transplantation Resistance of Type II Diabetes Mellitus Adipose-Derived Stem Cells Is Due to G6PC and IGF1 Genes Related to the FoxO Signaling Pathway
Source: Int J Mol Sci. 2021 Jun 19;22(12):6595. doi: 10.3390/ijms22126595 (PMC8235161; doi:10.3390/ijms22126595)
Supplement: Supplementary file 1 [file ijms-22-06595-s001.zip › Supplementary Figure 1.pdf]

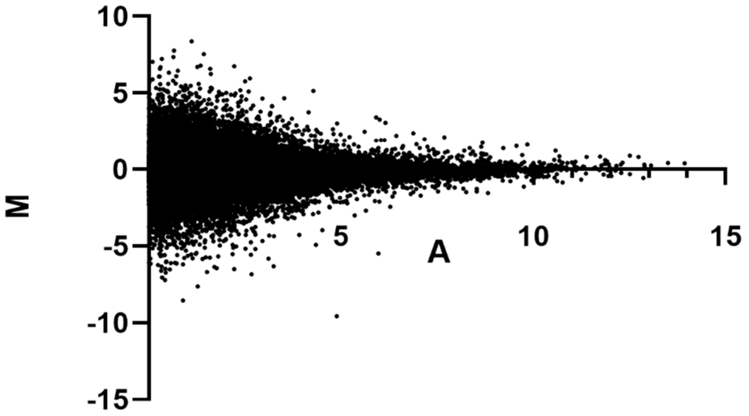

Supplementary Figure 1

The MA plot of the distribution of the gene expression data.

M value = log2(T2DM gene expression value)-log2(Normal gene expression value).

A value =| log2(T2DM gene expression value)+log2(Normal gene expression value)|/2.
